# Hypoalbuminemia predicts early postoperative complications following noninfectious revision total shoulder arthroplasty

**DOI:** 10.1007/s00590-024-04041-2

**Published:** 2024-07-08

**Authors:** Steven H. Liu, Patricia Cerri-Droz, Rachel A. Loyst, David E. Komatsu, Edward D. Wang

**Affiliations:** 1 Department of Orthopaedics, Stony Brook University, HSC T-18, Room 080, Stony Brook, NY 11794-8181 USA; 2 Department of Orthopaedic Surgery, Keck Medicine of USC, Los Angeles, USA

**Keywords:** Revision total shoulder arthroplasty, Total shoulder arthroplasty, Nutrition, Albumin, Hypoalbuminemia, Complications

## Abstract

**Purpose:**

This study investigates the association between preoperative hypoalbuminemia and 30-day postoperative complications following noninfectious revision total shoulder arthroplasty (TSA).

**Methods:**

The American College of Surgeons National Surgical Quality Improvement Program database was queried for all patients who underwent noninfectious revision TSA from 2015 to 2021. The study population was divided into two groups based on preoperative serum albumin: normal albumin (≥ 3.5 g/dL) and hypoalbuminemia (< 3.5 g/dL). Logistic regression analysis was conducted to investigate the relationship between preoperative hypoalbuminemia and postoperative complications.

**Results:**

Compared to normal albumin, hypoalbuminemia was independently associated with a significantly greater likelihood of experiencing any complication (odds ratio [OR] 3.26, 95% confidence interval [CI] 2.04–5.19; *P* < .001), sepsis (OR 9.92, 95% CI 1.29–76.35; *P* = .028), blood transfusions (OR 2.89, 95% CI 1.20–6.93; *P* = .017), non-home discharge (OR 2.88, 95% CI 1.55–5.35; *P* < .001), readmission (OR 3.46, 95% CI 1.57–7.58; *P* = .002), and length of stay > 2 days (OR 3.00, 95% CI 1.85–4.86; *P* < .001).

**Conclusions:**

Preoperative hypoalbuminemia was associated with early postoperative complications following revision TSA.

**Level of evidence:**

Level III; Retrospective Cohort Comparison; Prognosis Study.

## Introduction

Total shoulder arthroplasty (TSA) is a surgical treatment of osteoarthritis, inflammatory joint disease, complex proximal humerus fractures, and rotator cuff tear arthropathy, serving to restore functionality and alleviate pain. From 2011 to 2017, there was a 103.7% growth in the incidence of primary TSA procedures, largely due to its rapidly expanding indications, technological advancements, as well as the increased utilization of reverse TSA [1]. Accordingly, the annual volume of revision TSA has nearly tripled from 2012 to 2018 [2]. Epidemiological studies predict a continued rise in revision arthroplasty cases, which have greater rates of morbidity, mortality, and postoperative complications when compared to primary arthroplasty [2, 3].

Hypoalbuminemia is a laboratory finding underlying a variety of pathologic conditions including malnutrition, renal failure, and various inflammatory diseases [4]. Furthermore, hypoalbuminemia is a predictor of greater hospital length of stay (LOS), morbidity, and all-cause mortality following surgery [4, 5]. Therefore, hypoalbuminemia has shown utility in identifying high-risk patients in both orthopedic and other surgeries [6–9].

While the previous studies have investigated hypoalbuminemia as a risk factor for other orthopedic surgeries, none have studied hypoalbuminemia in the context of revision TSA. Given the high degree of morbidity in revision TSA cases, albumin status plays a crucial role in the preoperative workup of revision TSA patients. We hypothesized that patients with hypoalbuminemia would be at greater risk of early postoperative complications.

## Materials and methods

We queried the American College of Surgeons National Surgical Quality Improvement Program (ACS-NSQIP) database for all patients who underwent revision TSA from 2015 to 2021. This study was exempt from approval by our University’s Institutional Review Board because the NSQIP database is fully deidentified. Data in the NSQIP database are gathered from over 600 hospitals in the US by trained surgical clinical reviewers. The data are periodically reviewed to maintain high reliability.

**Fig. 1:**
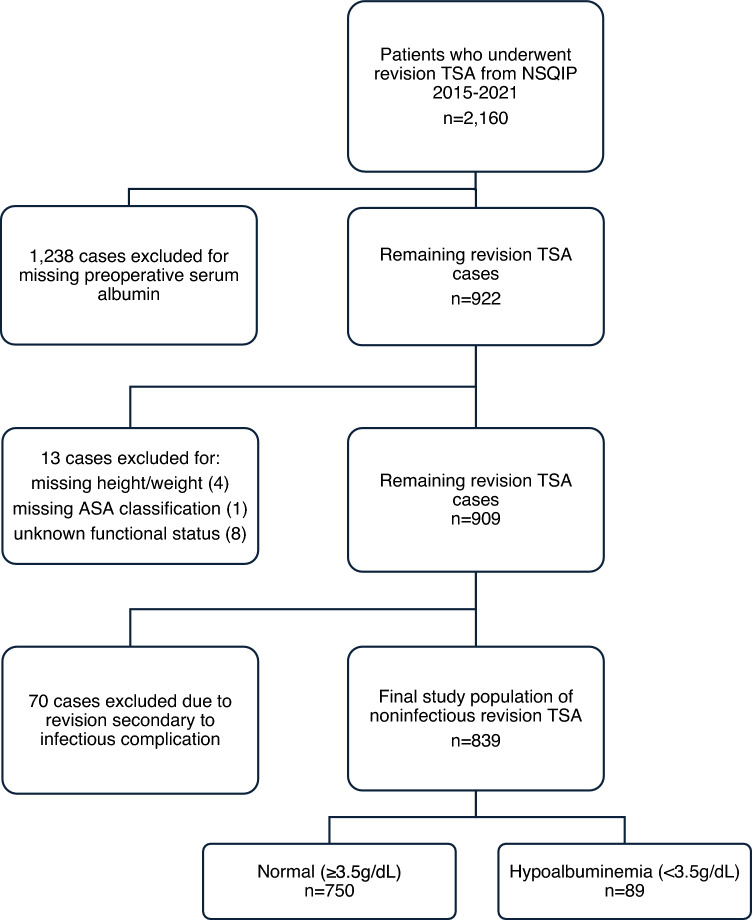
Case selection schematic. TSA, total shoulder arthroplasty; NSQIP, National Surgical Quality Improvement Program; ASA, American Society of Anesthesiologists

The current procedural terminology (CPT) codes 23,473–23,474 were used to identify 2160 patients who underwent revision TSA from 2015 to 2021 (Fig. 1). The NSQIP database inherently excludes all cases for patients younger than 18 years of age and all cases with primary admission related to trauma. The 1238 patients with missing preoperative serum albumin values were excluded. Next, 13 cases were excluded for missing height/weight, American Society of Anesthesiologists (ASA) classification, or functional health status. Finally, 70 cases were excluded due to revisions secondary to an infectious complication. Revisions due to infectious complications were excluded because the NSQIP database does not contain details regarding the nature of the infection (acute vs. chronic), which may significantly impact outcomes. The final patient population included in the study after exclusion criteria was 839, which were then separated into normal albumin (≥ 3.5 g/dL) and hypoalbuminemia (< 3.5 g/dL) groups.

Variables collected in this study included patient demographics, comorbidities, surgical characteristics, and 30-day postoperative complication data. Patient demographics included sex, age, body mass index (BMI), functional status, ASA classification, smoking status, and preoperative steroid use. Preoperative comorbidities included congestive heart failure (CHF), diabetes, hypertension, severe chronic obstructive pulmonary disease (COPD), bleeding disorders, and disseminated cancer. Thirty-day complications included the following: sepsis, septic shock, pneumonia, unplanned reintubation, urinary tract infection (UTI), cardiac arrest or myocardial infarction (MI), stroke, blood transfusions, deep vein thrombosis (DVT), pulmonary embolism (PE), on ventilator > 48 h, surgical space infection (SSI), wound dehiscence, acute renal failure, *Clostridioides difficile* (*C. diff*) infection, non-home discharge, readmission, unplanned reoperation, LOS > 2 days, and mortality.

Statistical analyses were performed with SPSS Software version 26.0 (IBM Corp., Armonk, NY, USA). Bivariate logistic regression was used to compare patient demographics and comorbidities between the two groups. Multivariate logistic regression, adjusted for all patient demographics and comorbidities significantly associated with hypoalbuminemia, was used to identify significant independent associations between hypoalbuminemia and postoperative complications. Odds ratios (OR) were reported with 95% confidence intervals (CI). The level of statistical significance was set at *P* < 0.05.

## Results

Compared to normal albumin, hypoalbuminemia was significantly associated with the female gender *(P* = 0.047), older age groups *(P* = 0.041), dependent functional status *(P* < 0.001), and ASA classification ≥ 3 *(P* = 0.005) (Table 1). Normal albumin was associated with comorbid diabetes *(P* = 0.007).

**Table 1:** Demographics and comorbidities of patients with normal preoperative serum albumin and hypoalbuminemia. Bold *P* values indicate statistical significance with *P* < .05

	Normal (≥3.5 g/dL)	Hypoalbuminemia (<3.5 g/dL)	
	Number (%)	Number (%)	*P* value
Overall	750 (100.0)	89 (100.0)	
*Sex*			.047
Female	388 (51.7)	56 (62.9)	
Male	362 (48.3)	33 (37.1)	
*Age*			.041
18–39	9 (1.2)	0 (0.0)	
40–59	142 (18.9)	12 (13.5)	
60–79	534 (71.2)	65 (73.0)	
≥80	65 (8.7)	12 (13.5)	
*BMI (kg/m^2)*			.544
<18.5	3 (0.4)	0 (0.0)	
18.5–24.9	127 (16.9)	12 (13.5)	
25–29.9	220 (29.3)	65 (73.0)	
≥30	400 (53.3)	12 (13.5)	
*Functional status prior to surgery*			<.001
Dependent	20 (2.7)	9 (10.1)	
Independent	730 (97.3)	80 (89.9)	
*ASA classification*			.005
≤2	274 (36.5)	19 (21.3)	
≥3	476 (63.5)	70 (78.7)	
*Smoker*			.933
No	672 (89.6)	80 (89.9)	
Yes	78 (10.4)	9 (10.1)	
*Steroid use*			.147
No	698 (93.1)	79 (88.8)	
Yes	52 (6.9)	10 (11.2)	
*Comorbidities*			
CHF	10 (1.3)	2 (2.2)	.497
Diabetes	143 (19.1)	5 (5.6)	.007
Hypertension	505 (67.3)	60 (67.4)	.987
COPD	52 (6.9)	9 (10.1)	.278
Bleeding disorder	28 (3.7)	5 (5.6)	.391
Disseminated cancer	1 (0.1)	1 (1.1)	.131
*Total operation time (minutes)*			.184
0–79	154 (20.5)	29 (32.6)	
80–128	309 (41.2)	28 (31.5)	
≥129	287 (38.3)	32 (36.0)	

BMI, body mass index; ASA, American Society of Anesthesiologists; CHF, congestive heart failure; and COPD, chronic obstructive pulmonary disease

Compared to normal albumin, hypoalbuminemia was significantly associated with a significantly greater likelihood of experiencing any complication *(P* < 0.001), sepsis *(P* = 0.033), blood transfusions *(P* = 0.003), non-home discharge *(P* < 0.001), readmission *(P* = 0.002), unplanned reoperation *(P* = 0.043), and LOS > 2 days *(P* < 0.001) (Table 2).

**Table 2:** Bivariate analysis of 30-day postoperative complications in patients with normal preoperative serum albumin and hypoalbuminemia. Bold *P* values indicate statistical significance with *P* < .05

	Normal (≥ 3.5 g/dL)	Hypoalbuminemia (< 3.5 g/dL)	
	Number (%)	Number (%)	*P* value
Any complication	171 (22.8)	48 (53.9)	< .001
Sepsis	2 (0.3)	2 (2.2)	.033
Septic shock	0 (0.0)	0 (0.0)	–
Pneumonia	2 (0.3)	1 (1.1)	.239
Unplanned reintubation	0 (0.0)	0 (0.0)	–
UTI	2 (0.3)	0 (0.0)	.999
Cardiac arrest or MI	2 (0.3)	1 (1.1)	.239
Stroke	0 (0.0)	0 (0.0)	–
Blood transfusions	20 (2.7)	8 (9.0)	.003
DVT	5 (0.7)	1 (1.1)	.633
PE	5 (0.7)	2 (2.2)	.145
On ventilator > 48 h	1 (0.1)	0 (0.0)	1.000
SSI	18 (2.4)	2 (2.2)	.929
Wound dehiscence	0 (0.0)	0 (0.0)	–
Acute renal failure	0 (0.0)	0 (0.0)	–
*C. difficile* infection	1 (0.1)	1 (1.1)	.131
Non-home discharge	47 (6.3)	19 (21.3)	< .001
Readmission	28 (3.7)	10 (11.2)	.002
Unplanned reoperation	20 (2.7)	6 (6.7)	.043
LOS > 2 days	119 (15.9)	36 (40.4)	< .001
Mortality	2 (0.3)	0 (0.0)	.999

UTI, urinary tract infection; MI, myocardial infarction; DVT, deep vein thrombosis; PE, pulmonary embolism; SSI, surgical site infection; and LOS, length of stay

After controlling for all associated patient demographic and comorbidity factors, an adjusted multivariate regression analysis was conducted. Compared to normal albumin, hypoalbuminemia was independently associated with a significantly greater likelihood of experiencing any complications (OR 3.26, 95% CI 2.04–5.19; *P* < 0.001), sepsis (OR 9.92, 95% CI 1.29–76.35; *P* = 0.028), blood transfusions (OR 2.89, 95% CI 1.20–6.93; *P* = 0.017), non-home discharge (OR 2.88, 95% CI 1.55–5.35; *P* < 0.001), readmission (OR 3.46, 95% CI 1.57–7.58; *P* = 0.002), and LOS > 2 days (OR 3.00, 95% CI 1.85–4.86; *P* < 0.001) (Table 3).

**Table 3:** Multivariate analysis of 30-day postoperative complications in patients with normal preoperative serum albumin and hypoalbuminemia. Bold *P* values indicate statistical significance with *P* < 05

	Hypoalbuminemia (< 3.5 g/dL)
	OR, *P* value (95% CI)
Any complication	3.26, < .001 (2.04–5.19)
Sepsis	9.92, .028 (1.29–76.35)
Blood transfusions	2.89, .017 (1.20–6.93)
Non-home discharge	2.88, < .001 (1.55–5.35)
Readmission	3.46, .002 (1.57–7.58)
Unplanned reoperation	2.53, .068 (0.94–6.87)
LOS > 2 days	3.00, < .001 (1.85–4.86)

LOS, length of stay

## Discussion

Using a large surgical database, we found that hypoalbuminemia was associated with a significantly greater likelihood of experiencing 30-day postoperative complications following any noninfectious revision TSA. Compared to normal albumin status, hypoalbuminemia was independently associated with a significantly greater likelihood of experiencing any complications, sepsis, blood transfusions, non-home discharge, readmission, and LOS > 2 days.

Hypoalbuminemia, defined as serum albumin ≤ 3.5 g/dL, is often observed in chronically ill patients. The pathogenesis of hypoalbuminemia is partly due to increased vascular permeability resulting from the upregulation of inflammatory processes in individuals in a diseased state [10]. While it can exist acutely, hypoalbuminemia is most frequently the result of persistent disease, with its severity correlating to a worsening degree of trauma, health status (obesity, smoking, and diet), chronic disease, cancer, etc. [4–11]. Revision TSA possesses a significantly elevated complication rate, reaching up to 38%, a rate that may be exacerbated by the presence of preoperative hypoalbuminemia [12].

We found that 86.5% of patients with hypoalbuminemia were age 60 or older. This was consistent with the literature showing that low serum albumin levels are more prevalent in hospitalized patients aged 65 and above [13]. Furthermore, we found that patients with hypoalbuminemia were more likely to be female, exhibit dependent functional status, and have a greater ASA classification. These demographic trends align with those seen in patients with hypoalbuminemia undergoing proximal humerus fracture fixation [7].

Reduced albumin levels compromise its function as an antioxidant, extracellular transport molecule, and supplier of amino acids for synthesis reactions within the body. Postoperatively, this imbalance may manifest as poor healing and surgical site infections that have been observed in spinal and total joint replacement surgeries in patients with hypoalbuminemia, or more extensive infectious complications [14, 15]. The patients in our study with hypoalbuminemia were predisposed to postoperative complications and were at a nearly tenfold risk of sepsis (OR 9.92). The already heightened risk of sepsis associated with revision TSA is, therefore, compounded by the systemic vulnerability to infection associated with hypoalbuminemia [16]. Additionally, we found that patients with hypoalbuminemia had a nearly threefold risk of requiring a postoperative blood transfusion (OR 2.89) following revision TSA. This pattern is similar to that of primary TSA, in which hypoalbuminemia was found to have a 2.5-fold greater risk of requiring a blood transfusion [17]. While a causal relationship cannot be established, hypoalbuminemia may reflect a generally poorer health status and a reduced ability to heal wounds, making these patients more susceptible to bleeding complications.

Along with a globally increased postoperative complication rate, patients with hypoalbuminemia were also more likely to experience complications related to adverse hospital metrics. Our study revealed that patients with hypoalbuminemia were readmitted and experienced LOS > 2 days at a greater rate, findings consistent with a study of primary total knee and hip arthroplasties [15]. In addition, there was a nearly threefold increase in the odds of patients with hypoalbuminemia being discharged to a non-home location (OR 2.88). These results could negatively impact both patient experience and outcomes, as well as have adverse financial implications for the hospital system.

There are several limitations to the current study, many of which arise from use of a large database. The ACS-NSQIP database provides an extensive and representative population for analysis and is regularly maintained for accuracy. However, information on patients is limited by the 30-day time frame captured by the database. This excludes occurrences that may take place following this period, including issues with functionality, range of motion, and patient satisfaction. Furthermore, we are limited by inpatient and outpatient information only, as it does not provide data on procedures done in centers not affiliated with a hospital. In addition, we were unable to control for the indications for revision arthroplasty. This may affect the findings of this study since different indications of revision TSA are associated with varying degrees of risk. It is also important to acknowledge that a notable portion of our study population was excluded for missing albumin levels. This may indicate that patients already seen as higher risk are having serum albumin levels ordered, which may influence the rates of complication observed. Nevertheless, this study encompasses a large population and demonstrates a greater risk of early postoperative complications in patients with hypoalbuminemia undergoing revision TSA. Depending on etiology, low albumin levels may be a modifiable risk factor, which should encourage future studies focusing on the utility of preoperative or postoperative intervention in reducing rates of complications in revision TSA.

## Conclusion

Preoperative hypoalbuminemia was independently significantly associated with a greater rate of early postoperative complications following noninfectious revision TSA. A better understanding of preoperative hypoalbuminemia as a risk factor for postoperative complications following revision TSA may allow surgeons to better select surgical candidates and correct modifiable risk factors before revision TSA to improve surgical outcomes.
